# Clinical experience with near-infrared ray catheter, a fluorescent ureteral catheter, on laparoscopic surgery for colon diverticulitis: A case report

**DOI:** 10.1097/MD.0000000000026085

**Published:** 2021-05-28

**Authors:** Wataru Osumi, Masashi Yamamoto, Kohei Taniguchi, Shinsuke Masubuchi, Hiroki Hamamoto, Masatsugu Ishi, Keisuke Izuhara, Keitaro Tanaka, Junji Okuda, Kazuhisa Uchiyama

**Affiliations:** a Department of General and Gastroenterological Surgery; b Translational Research Program; c Cancer Center, Osaka Medical and Pharmaceutical University, Takatsuki, Osaka, Japan.

**Keywords:** case report, colovesical fistulas secondary to colon diverticulitis, laparoscopic surgery, NIRC fluorescent ureteral catheter

## Abstract

**Rationale:**

As the world's population ages, the number of surgical cases of colovesical fistulas secondary to colon diverticulitis is also expected to increase. The key issue while performing laparoscopic surgery for these fistulas is the avoidance of iatrogenic ureteral injury. There are no reports of Near-infrared Ray Catheter being used in surgery for diverticulitis, which is one of the diseases with the highest risk of ureteral injury. We present a case of a male patient with colovesical fistulas secondary to sigmoid colon diverticulitis who underwent laparoscopic surgery with visualization of the ureter using a new surgical technique in laparoscopic surgery.

**Patient's concern:**

An 82-year-old man presented to our urological department with general fatigue and air and fecal matter in the urine.

**Diagnoses:**

Cystography showed delineation of the sigmoid colon. Abdominal computed tomography findings revealed multiple sigmoid colon diverticula with thickened walls as well as large stones and a small amount of air in the bladder. He was diagnosed with a urinary tract infection with colovesical fistulas and bladder stones due to sigmoid diverticulitis.

**Interventions:**

After the creation of a transverse colostomy, we scheduled a laparoscopic anterior resection and cystolith removal.

**Outcomes:**

Severe inflammatory adhesions around the sigmoid colon and a high risk of ureteral injury were expected preoperatively. After induction of anesthesia, we inserted a Near-infrared Ray Catheter, a fluorescent ureteral catheter, which allowed us to easily identify and visualize the ureter in real-time. This allowed bowel dissection without concerns of ureteral injury. The operative time for the gastrointestinal part of the procedure was 150 minutes, and the patient was in a good general condition after the operation and was discharged on postoperative day 7.

**Lessons:**

The course of the ureter was easily and quickly identified by the green fluorescence from the ureteral catheter during laparoscopic surgery for fistulas associated with diverticulitis, where severe inflammation and dense fibrosis were present. Our technique is an easy and feasible approach that provides real-time urethral navigation during surgery for colovesical fistulas secondary to colon diverticulitis.

## 1 Introduction

As the world's population ages, the number of surgical cases of colovesical fistulas secondary to colon diverticulitis (CFD) is expected to increase.^[1–3]^ Laparoscopic surgery is widely used in gastrointestinal surgery because of its advantages, which are as follows: less bleeding, faster recovery of intestinal peristalsis, less pain, and quicker return to society. However, in cases of CFD with severe inflammation, even though many authors have reported the usefulness of laparoscopy, the procedure is still technically challenging due to its high conversion rate from laparoscopic to open surgery.^[1]^ One of the reasons for this is the difficulty in identifying the ureter due to severe inflammation.^[4]^

In recent years, with the technological development of endoscopic surgery and surgical assistive robots, indocyanine green (ICG) technology has been used to visualize blood flow and the lymphatic system^[5–7]^ and has been applied in intraoperative ureteral navigation.^[8–10]^ The Near-infrared Ray Catheter (NIRC; Nippon Covidien, Ltd., Tokyo, Japan), which was recently approved for clinical use in Japan, is a fluorescent ureteral catheter that emits fluorescence under near-infrared light. When irradiated with near-infrared light, the ureter is fluorescently illuminated and the course of the ureter is clearly visualized. Therefore, the NIRC is very useful for performing real-time navigation in endoscopic systems equipped with a far-infrared mode in laparoscopic colorectal surgery. The NIRC has been reported to be used in colorectal cancer surgery.^[11]^ However, there are no reports of NIRC being used in surgery for diverticulitis, which is one of the diseases with the highest risk of ureteral injury.

Herein, we report a case of a male patient with colovesical fistulas secondary to sigmoid colon diverticulitis who underwent laparoscopic surgery and visualization of the ureter using the NIRC, which was very useful.

## 2 Case presentation

**Figure 1:**
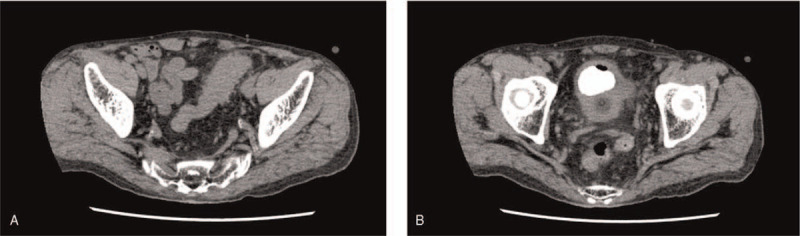
The representative images of abdominal computed tomography (CT). (A) Abdominal computed tomography findings show multiple sigmoid colon diverticula with thickened walls. (B) Large stones and a small amount of air are found in the bladder, suggesting the presence of a colonic cystic fistula.

An 82-year-old man presented to our urological department with general fatigue and air and fecal matter in the urine. He had been receiving treatment for kidney and bladder stones for 30 years and had also undergone open kidney lithotripsy and extracorporeal shock wave lithotripsy. He had also been hospitalized previously for conservative treatment of sigmoid diverticulitis. During the presentation, physical examination showed only severe left lower abdominal tenderness. He had a very high fever, with otherwise normal vital signs. Blood test results revealed that his C-reactive protein level was elevated by 5.07 mg/dL. Other blood test results were within the normal range. Cystography showed delineation of the sigmoid colon. Abdominal computed tomography findings revealed multiple sigmoid colon diverticula with thickened walls (Fig. 1A) as well as large stones and a small amount of air in the bladder (Fig. 1B). He was diagnosed with a urinary tract infection with colovesical fistulas and bladder stones due to sigmoid diverticulitis.

**Figure 2:**
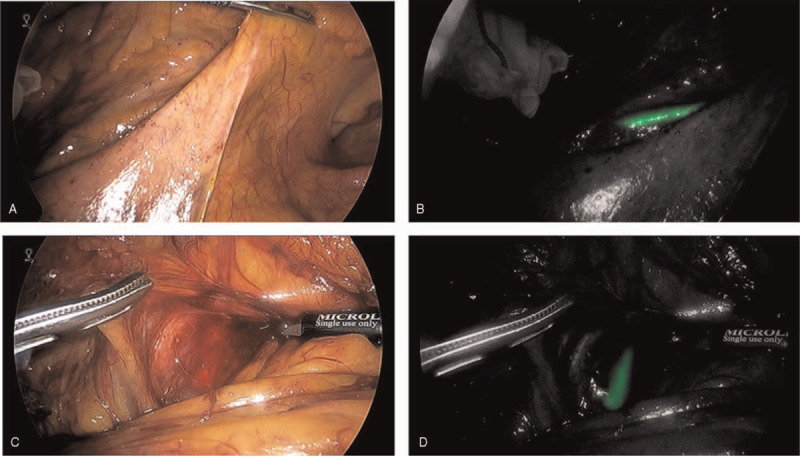
The representative intraoperative images. Intraoperative images show the ureter under the mesentery of the sigmoid colon before dissection (A) under standard light and (B) with the use of near-infrared laser fluorescence technology. Before dissection of the peritoneum, the ureter is clearly identifiable. An intraoperative image in the presence of microbleeding (C) under standard light and (D) with the use of near-infrared laser fluorescence technology. The course of the ureter is easily seen despite the presence of microbleeding.

Initially, a laparoscopic transverse colostomy was performed to manage the urinary tract infection. Infection control by colostomy resulted in the improvement of his general condition, and he requested closure of the colostomy in the future. Three months later, laparoscopic anterior resection and cystolith removal were conducted. He had a high risk for severe inflammatory adhesions around the sigmoid colon and ureteral injury. Hence, we decided to use the NIRC also. After induction of anesthesia, the patient was placed in a lithotomy position. A guidewire was inserted from the ureter into the renal pelvis under cystoscopic and fluoroscopic guidance; then, the NIRC was inserted into the renal pelvis, and the guidewire was removed by the urology team. Laparoscopic surgery and fluorescence observation were performed with the 5 ports method using the VISERA ELITE 2 system (Olympus, Tokyo, Japan). As expected, the entire sigmoid colon and surrounding tissue were highly thickened and adherent to the surrounding peritoneum and retroperitoneal subfascia. We proceeded with the dissection of the sigmoid colon and fistula. Ureteral navigation using the NIRC was safe and easy to perform during bowel dissection close to the ureter, with its course clearly identified (Fig. 2A–D). Additional mobilization of the splenic flexure and upper rectum was performed to release the anastomotic hyperintensity. The operative time for the gastrointestinal part of the procedure was 150 minutes, with minimal blood loss. Although there was no visually obvious full-layer defect in the bladder wall, two 3–0 PDSII (Johnson & Johnson, New Brunswick, New Jersey) sutures were used to close the serosal membrane defect of the bladder. After bowel dissection and anastomosis, the urology team performed laparoscopically assisted cystolith removal. The total operative time, including the later cystotomy, was 318 minutes. We, thus, identified the ureter and safely performed laparoscopic anterior resection for sigmoid colon diverticulitis with cystocolon fistulas.

The patient's postoperative course was uncomplicated, and he was discharged from the hospital on postoperative day 7. Approximately 3 months later, when the transverse colostomy was closed, he was still in remission.

## 3 Discussion

In the present report, we describe a case of a male patient with colovesical fistulas who had severe inflammation and high-density fibrosis around the ureter due to chronic diverticulitis, and the employment of NIRC enabled real-time ureteral navigation for easy and safe laparoscopic surgery.

Recently, a large cohort study involving 111 laparoscopic surgical cases of CFD was reported.^[4]^ The conversion rate from laparoscopic to open surgery was high (34.7%), with one of the main reasons being difficulty in identification of the ureter due to severe inflammation and dense fibrosis, resulting in concerns regarding the safe completion of the operation. Ureteral injury is a relatively rare, but serious, complication of colorectal surgery. Previous pelvic surgery and inflammatory bowel disease are considered to be risk factors.^[12]^ Preoperative ureteral stenting is widely used in cases of threatened ureteral injury. A previous study reported that, in the laparoscopic surgery for CFD, preoperative ureteral stenting was performed approximately 3.3 times more frequently compared to that for malignant tumors.^[13]^ Ureteral injury experts have regarded ureteral stenting as useful in detecting intraoperative ureteral injury, as the stent is visible at the time of the injury. However, it does not decrease the incidence of ureteral injury.^[14]^ The NIRC enhances real-time ureteral delineation in endoscopic systems equipped with a far-infrared mode; this is simple and easy to use with only the mode conversion of the endoscope. The fluorescence response can be seen under the mesentery of the sigmoid colon before dissection and in the presence of microbleeding, with clear identification of the ureter (Fig. 2B and 2D). This fluorescent stent is easy to use and powerful in visualizing the ureter, and it may contribute to reducing the risk of ureteral injury and safer laparoscopic surgery in CFD, where the conversion rate to open surgery and the risk of ureteral injury are higher compared to those of malignant tumors.

Although intraoperative ureteral navigation using ICG has been reported in the past,^[8,10]^ it is not widely used at present. It has been reported that the ureter can be clearly visualized by injecting ICG into the ureteral stent, but this has not yet been approved by the authorities, which is expected to prevent it from being employed generally.^[8]^ However, the NIRC has already been approved for clinical use and is expected to be widely used in the near future.

The insertion of the NIRC is similar to conventional ureteral implantation. The urology team, who were unfamiliar with the NIRC, performed the insertion procedure smoothly in 12 minutes. Previous studies have reported the problem of prolonged total operative time with stent insertion.^[15,16]^. Although the NIRC also prolongs the total operative time, in severe inflammation cases where more time is spent intraoperatively to check the course of the ureter, visualization of the ureter by using the NIRC can help in shortening the operative time. Above all, we believe that the advantage of performing surgical treatment safely under direct vision is imperative in a complicated case such as ours with a high degree of adhesions. Given that we only reported our experience with a single case-experience, we plan to accumulate more cases in the future to confirm our findings.

In conclusion, we believe that our technique is an excellent method for performing laparoscopic surgeries as a treatment for fistulas and diverticulitis with severe inflammation, as in the present case where there was a high risk of ureteral injury because it is simple to use and allows us to confirm the ureteral motion.

## Acknowledgments

The authors are grateful for the cooperation of the personnel from the Department of Urology, Osaka Medical College, who were involved in the ureteral catheter procedure. The authors also thank Editage (www.editage.jp) for English language editing.

## Author contributions

**Conceptualization:** Wataru Osumi.

**Data curation:** Wataru Osumi, Masashi Yamamoto, Shinsuke Masubuchi, Hiroki Hamamoto, Masatsugu Ishi, Keisuke Izuhara, Keitaro Tanaka, Junji Okuda.

**Investigation:** Wataru Osumi, Kohei Taniguchi, Shinsuke Masubuchi, Hiroki Hamamoto, Masatsugu Ishi, Keisuke Izuhara, Keitaro Tanaka, Junji Okuda.

**Project administration:** Wataru Osumi, Masashi Yamamoto.

**Supervision:** Kazuhisa Uchiyama.

**Writing – original draft:** Wataru Osumi.

**Writing – review & editing:** Wataru Osumi, Kohei Taniguchi.
